# Examining brain structures associated with dispositional envy and the mediation role of emotional intelligence

**DOI:** 10.1038/srep39947

**Published:** 2017-02-08

**Authors:** Yanhui Xiang, Sasa Zhao, Hanlin Wang, Qihan Wu, Feng Kong, Lei Mo

**Affiliations:** 1 Center for Study of Applied Psychology, South China Normal University, Guangzhou, Guangdong, China; 2 School of Psychology, South China Normal University, Guangzhou, Guangdong, China; 3 Guangdong Key Laboratory of Mental Health and Cognitive Science, South China Normal University, Guangzhou, Guangdong, China; 4 School of Psychology, Shaanxi Normal University, Xi’an, Shaanxi, China

## Abstract

Dispositional envy is distinguished by definition and neurally from episodic envy. While the neural correlates of episodic envy have been evaluated by specific tasks in previous studies, little is known about the structural neural basis of dispositional envy. In this study, we investigated the structural neural basis of dispositional envy underlying individual differences across two independent samples comprising a total of 100 young healthy adults. Firstly, 73 subjects’ data (sample 1) was analyzed, and we assessed the association between regional gray matter volume (rGMV) and dispositional envy using voxel-based morphometry (VBM). Furthermore, we explored the role of emotional intelligence in the association between GMV and dispositional envy. VBM indicated that dispositional envy was positively correlated with GMV in the left dorsolateral prefrontal cortex (DLPFC) and superior temporal gyrus (STG). We also found that emotional intelligence partially mediated the association between DLPFC volume and dispositional envy. These results were replicated in another independent sample (Sample 2, n = 27). These results provide the first evidence that dispositional envy exhibits a structural neural correlation with the DLPFC and STG, and give a neutral explanation for why individuals with high emotional intelligence exhibit less envy.

Envy is a natural yet powerful human emotion^1^ that arises “when a person lacks another’s superior quality, achievement, or possession and either desires it or wishes that the other lacked it”^2^. Aristotle described envy as an irrational, unpleasant feeling accompanied by a sense of inferiority, anxiety, or resentment^3^. Previous studies have explored the neural correlates of episodic envy using specialized tasks^4^,^5^. However, little is known regarding the neural basis of dispositional envy. Exploring the neural correlates of dispositional envy may provide a neurological explanation for why some people tend to feel more envious than others in comparable social situations. Some researchers have suggested that episodic and dispositional envy share a number of common characteristics, though recent work utilizing a state-versus-trait framework has highlighted several key differences between chronic feelings (dispositional) and occasional experiences (episodic) of envy^6^,^7^. Therefore, in the present study, we aimed to investigate the structural neurological basis of dispositional envy using voxel-based morphometry (VBM). Furthermore, we will elucidate the role of emotional intelligence in predicting dispositional envy.

Neuroimaging and neurobiological studies have identified a major role the prefrontal cortex (PFC) play in the processing of envy with particular emphasis on the medial prefrontal cortex (MPFC). Takahashi *et al*.^4^ demonstrated that activation of the MPFC, especially the dorsal anterior cingulate cortex (dACC), is positively correlated with the degree of envy experienced during a functional magnetic resonance imaging (fMRI) task. Furthermore, Shamay-Tsoory *et al*.^5^ observed that patients with MPFC injuries were unable to identify envy looks. The results of these studies indicate that the PFC (including the MPFC and ACC) may be extensively involved in the processing of episodic envy. Feelings of episodic envy as well as associated regions of activation tend to be largely task- and situation-specific; however, dispositional envy is thought to reflect an individual’s tendency to experience envy across a variety of tasks and situations^7^,^8^. Previous studies have conjectured that the experience of episodic envy involves two principle components: the emotional experience and the cognitive appraisal process^6^. Dispositional envy, on the other hand, is associated with stable/chronic feelings of inferiority and ill will^2^,^9^, which may significantly affect the aforementioned cognitive appraisal process, thereby giving rise to increased feelings of envy. Therefore, we hypothesized that dispositional envy would not only exhibit common regions of activation with episodic envy but would also be associated with activity in more regions involved in emotion regulation.

Previous studies have indicated that several areas of the PFC (e.g., the dorsolateral prefrontal cortex [DLPFC] and ventrolateral prefrontal cortex [VLPFC]) play an important role in the integration of emotion and cognition^10^,^11^,^12^. For example, MRI studies have revealed abnormalities in the DLPFC, VLPFC, and orbitofrontal cortex (OFC) in patients with psychiatric conditions^13^,^14^. Furthermore, a large body of work supports the conclusion that alterations in the DLPFC and VLPFC lead to abnormal emotional expression, poor interpersonal skills, and deficits in self control^15^,^16^,^17^. In addition, some studies using transcranial direct current stimulation have directly demonstrated that activity in the left DLPFC negatively affects emotional control and negative emotion processing^18^. Results of these studies demonstrate that several regions of the PFC are widely implicated in emotional and cognitive processing. Some voxel-based morphometry (VBM) studies have also explored the correlation between regional gray matter volume (rGMV) in the PFC and emotional regulation: Kong *et al*.^19^ observed a significant negative correlation between rGMV in the DLPFC and social well-being in young healthy adults. Similarly, Takeuchi *et al*.^20^ observed a significant negative correlation between rGMV in the rostrolateral/dorsomedial PFC and quality of life in young healthy adults—regions typically associated with the regulation of negative emotions. These results suggest that increased rGMV in some regions of the PFC, especially in the DLPFC or DMPFC, may be associated with decreased abilities for emotional regulation in young healthy adults. Therefore, we hypothesized that rGMV in the DLPFC and DMPFC might be a significant positive predictor of dispositional envy in young healthy adults.

In addition to the PFC, dispositional envy may also recruit the activation of regions related to the perception of emotions or intentions, such as the temporal gyrus (TG). Envy is a negative emotion experienced during social comparisons or interpersonal communication^7^; that is, envy requires the perceived appraisal of self-inferiority within a specific social context. Therefore, the ability to perceive and interpret the characteristics, disposition, and intentions of other people is critical to the experience of envy. Previous studies have demonstrated that the TG is widely correlated with emotional perception. For example, Carr *et al*.^21^ indicated that the superior temporal gyrus (STG) and inferior frontal gyrus (IFG) are regions critical to the comprehension of a person’s intention during interpersonal communication. Wicker *et al*.^22^ indicated that the STG is selectively activated during analysis of emotions via eye contact with others. In a meta-analysis, Gallagher *et al*.^23^ concluded that the bilateral temporal pole is the foundation for speculating on or perceiving the psychological state of others. In conclusion, the TG and especially the STG may play an important role in the processing of envy. Therefore, we predicted that the experience of dispositional envy would be positively correlated with activation of the STG significantly.

On the premise that dispositional envy is associated with a chronic sense of inferiority developed over a long period of time, we also hypothesized the involvement of regions associated with emotional perception and regulation. Emotional intelligence is defined as a reflection of one’s ability to perceive, use, and regulate the emotions of oneself and others^24^. Among the functions of emotional intelligence, regulation of emotion is critical for the expression of various negative emotions. Accordingly, we speculated that individuals with higher emotional intelligence are more likely to possess a better capacity for perceiving and reasoning their own emotions as well as those of others. Dispositional envy may exhibit a close negative correlation with emotional intelligence, and the persistent experience of envy may be linked to abnormalities in emotional perception and reasoning abilities. On this premise, we also hypothesized that emotional intelligence mediated the correlation between dispositional envy and activity in certain brain regions.

In conclusion, the present study aimed to explore the relationships of different brain structures with dispositional envy. A voxel-based morphometry (VBM) method was used to identify neural correlates of individual differences in dispositional envy. Moreover, we speculated that emotional intelligence could be used to predict dispositional envy, and thus we examined whether emotional intelligence mediated the relationships between brain structures and dispositional envy.

## Results

Means, standard deviations (SDs), skewness, and kurtosis for Dispositional Envy Scale (DES) and Wong and Law Emotional Intelligence Scale (WLEIS) scores are presented in Table 1. The kurtosis and skewness of all scores ranged from −1 to 1, thus indicating the normality of the data^25^. In addition, DES scores were not significantly correlated with participants’ age (r = −0.170, *p* = 0.152) or total GMV (r = 0.031, *p* = 0.797).

**Figure 1:**
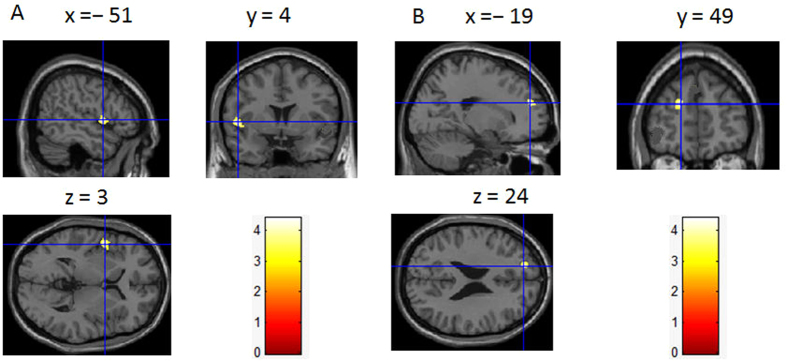
Brain regions exhibiting positive correlation with dispositional envy. Regional gray matter volumes (GMV) in the superior temporal gyrus (STG) (**A**) and the dorsolateral prefrontal cortex (DLPFC) (**B**) were positively correlated with Dispositional Envy Scale (DES) scores. These correlations were based on whole-brain analyses.

**Figure 2:**
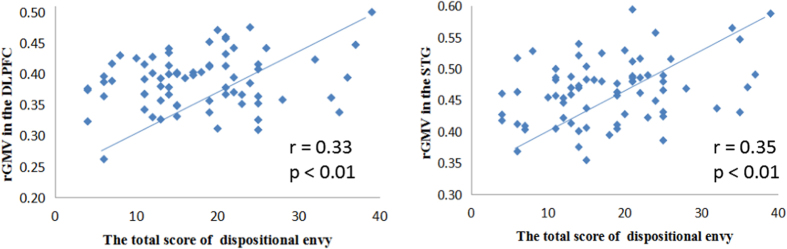
DES scores were significantly correlated with rGMV in the dorsolateral prefrontal cortex (DLPFC) and superior temporal gyrus (STG) in Sample 1. DES: Dispositional Envy Scale; rGMV: regional gray matter volume.

In order to determine the neural correlates of dispositional envy, we analyzed the correlation between DES scores and GMV for each voxel across the whole brain. After controlling for age, sex, and global GMV, the results of this analysis revealed positive correlations of dispositional envy with two clusters: the STG [ MNI coordinate: −52, 4, 3; r = 0.44, t = 4.123; *p* < 0.001], another cluster is the DLPFC extend to DMPFC (including super frontal gyrus and middle frontal gyrus in the atlas of AAL), [MNI coordinate: −19, 49, 24; r = 0.46, t = 4.400; *p* < 0.001] (see Table 2, Figs 1 and 2). No other correlations were observed.

After identifying potential neural correlates of dispositional envy, we further investigated the effect of emotional intelligence on the relationship between dispositional envy and GMV in the relevant regions (i.e., STG, DLPFC). We first performed a correlation analysis of WLEIS and DES scores, the results of which suggested that emotional intelligence is significantly correlated with dispositional envy (r = −0.579, *p* < 0.001, see Table 1). We then examined whether rGMV of regions related to dispositional envy (i.e., STG, DLPFC) could predict emotional intelligence. The results of this analysis indicated that rGMV in the DLPFC (r = −0.245, *p* = 0.036), but not in the STG (r = −0.186, *p* = 0.114), was significantly correlated with emotional intelligence, suggesting a close association among emotional intelligence, rGMV in the DLPFC, and dispositional envy.

**Figure 3:**
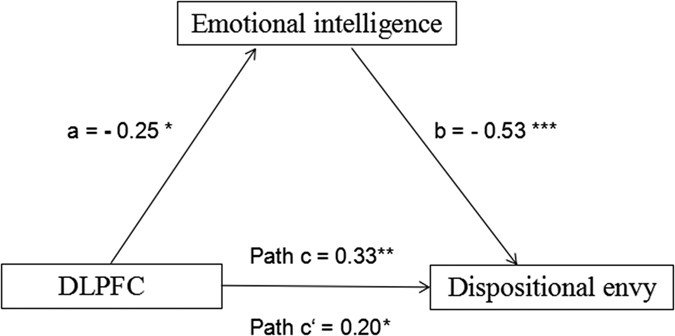
Emotional intelligence mediates the relationship between brain structure and dispositional envy. Paths a, b, c, c’ all refer to standard regression coefficients. Path a: rGMV in the DLPFC significantly predicts emotional intelligence. Path b: Emotional intelligence significantly predicts dispositional envy. Path c: rGMV in the DLPFC significantly predicts dispositional envy. Path c’: After controlling for emotional intelligence, rGMV in the DLPFC predicts dispositional envy. rGMV: regional gray matter volume; DLPFC: dorsolateral prefrontal cortex.

To investigate whether GMV in the DLPFC influenced dispositional envy via emotional intelligence, a mediation analysis was conducted (see Fig. 3). First, the total effect of the relationship between rGMV and dispositional envy was quantified without controlling for emotional intelligence. The results of this initial analysis demonstrated that rGMV in the DLPFC is a significant predictor of DES score (β = 0.334, *p* = 0.004). Second, after controlling for emotional intelligence, we repeated this analysis and observed a reduced effect of rGMV in the DLPFC on DES scores (β = 0.204, *p* = 0.040), and that emotional intelligence remained a significant predictor of dispositional envy (β = 0.528, *p* < 0.001). A subsequent bootstrap simulation (n = 5000) confirmed that this reduction was statistically significant [95% confidence interval = (3.03, 26.48)]. These data suggested that emotional intelligence mediated the relationship between DLPFC and dispositional envy.

**Figure 4:**
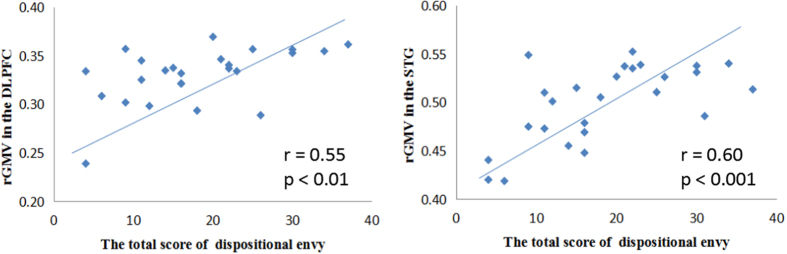
DES scores exhibit significant correlation with rGMV in the dorsolateral prefrontal cortex (DLPFC) and superior temporal gyrus (STG) in Sample 2. DES: Dispositional Envy Scale; rGMV: regional gray matter volume.

In order to assess the validity of the results, an additional 27 samples were utilized. We extracted rGMV data from the STG and DLPFC from an independent sample of 27 participants (Sample 2) based upon our initial analysis of regions associated with dispositional envy in the 73 participants of Sample 1. The results of this analysis revealed that rGMV in the DLPFC (r = 0.548, p = 0.003) and STG (r = 0.596, *p* < 0.001) were significantly correlated with dispositional envy, thus replicating the results obtained from Sample 1 (see Fig. 4). We further observed that emotional intelligence was significantly correlated with rGMV in the DLPFC (r = −0.494, *p* = 0.009) and dispositional envy (r = −0.697, *p* < 0.001), but not with rGMV in the STG (r = −0.209, *p* = 0.295) in Sample 2. Furthermore, using a mediation analysis, we again investigated the mediated effect of emotional intelligence on the relationship between rGMV in the DLPFC and dispositional envy. The results of this analysis revealed this mediating effect was significant (95% confidence interval = [5.22, 47.42], *p* < 0.05), again replicating the results obtained from another independent sample.

## Discussion

To the best of our knowledge, the present study marks the first attempt to investigate the neural correlates of individual differences in dispositional envy utilizing VBM analysis. The results of our analyses indicate that dispositional envy is positively correlated with rGMV in the DLPFC and STG, a result that proved to be stable and repeatable in a completely independent sample. The present study provides the first evidence regarding specific brain structures underlying individual differences in dispositional envy.

We observed a positive association between rGMV in the DLPFC and dispositional envy, which is consistent with the results of previous studies that have investigated the role of the DLPFC in emotional regulation. Hooker and Knight^26^ indicated that the DLPFC plays a crucial role in emotion regulation, while Heller *et al*.^27^ confirmed that the DLPFC is important for the processing of emotional stimuli. In addition, Luo *et al*.^28^ observed increased resting-state functional activity in the DLPFC of unhappy people. The results of these studies indicate that the DLPFC functions to regulate the balance between negative and positive emotions. Furthermore, previous VBM studies have demonstrated a correlation between rGMV in the DLPFC and the ability to regulate positive or negative emotions: Kong *et al*.^19^ observed a significant negative association between rGMV in the DLPFC and social well-being in young healthy adults; Takeuchi *et al*.^20^ found a significant negative correlation between rGMV in some regions of the DLPFC and quality of life in young healthy adults. That is, the higher level of happiness and quality of life people have, the higher ability to regulate negative emotions they exhibit. These results suggest that larger rGMV in the DLPFC may be associated with more negative emotions, thus leading to lower levels of happiness or quality of life in young healthy adults. Therefore, envy, as the synthesis of multiple negative emotion (such as inferiority, fear, anxiety, etc), may show the significant positive association with rGMV in DLPFC. In addition, previous studies also demonstrated that some regions of the DLPFC were related closely to evaluate fairness of observed behavior^29^,^30^,^31^, while others have suggested that injustice is the core characteristic for envy^32^,^33^,^34^. Thus, the association between rGMV in the DLPFC and envy may imply a certain relationship between rGMV of the DLPFC and injustice. Furthermore, some studies show the intracortical myelination and synaptic pruning that occur in some regions during development may be related to increased efficiency of cognitive processes^35^,^36^,^37^, including the process of regulating emotion. Therefore, the larger rGMV of DLPFC in the present study may mean lower efficiency in regulating emotions, thus leading to higher level of envy.

In addition, we revealed that emotional intelligence effectively mediated the relationship between rGMV in the DLPFC and dispositional envy in Sample 1, which also was replicated in Sample 2. More specially, we found the rGMV of DLPFC could predict significantly EI negatively. According to a previous study, EI can be defined as the accurate appraisal and expression of emotion in oneself and others^38^, which is a reflection of one’s ability to perceive, use, and regulate the emotion of oneself and others^24^. That is, higher EI is associated with higher emotional regulation. Furthermore, it is well known that the DLPFC plays an important role in emotional regulation^10^,^11^,^12^,^27^. Therefore, the significant negative association between rGMV of the DLPFC and EI suggest that larger rGMV in the DLPFC is indicative of reduced ability for emotional regulation, thus leading to higher level of dispositional envy. Basing on the discussion above, it is very easy to understand why the EI play the mediation role in the association between rGMV of the DLPFC and dispositional envy.

Additionally, it should be noted that the DLPFC also included portions of the MPFC in the present study. Previous studies have investigated that the MPFC is associated with self-evaluation^39^,^40^. An fMRI study by Takahashi *et al*.^4^ demonstrated a link between envy and activation of the DMPFC, especially the dACC. A VBM analysis by Vogt^41^ also revealed that individuals with increased rGMV in the DMPFC (including the dACC) might experience increased levels of negative emotion associated with self-evaluation. As negative self-evaluation is one of the defining characteristics of envy^42^,^43^, this result may indicate that those with higher tendency for dispositional envy may experience increased negative self-evaluation.

We further observed that dispositional envy was correlated with rGMV in the STG. The STG plays an important role in social cognition with regard to the perception and comprehension of the emotions/intentions of others^21^,^22^,^23^. An fMRI study by Perry *et al*.^44^ demonstrated that the STG was active when participants were required to provide empathic responses to sentences describing positive and negative situations of others. Morelli and Lieberman^45^ also demonstrated that the pSTG was active when participants were instructed to empathize with the situations of others (e.g., graduation or bereavement). The results of these studies indicate that the STG is closely linked to perceive, understand, and reason the emotions or intentions of others. As envy is derived from social comparisons with self-relevant individuals^46^, interpersonal perception is key for evoking feelings of envy. Some studies have demonstrated that the perception of high or low feelings of control^47^,^48^ and of justice or injustice^49^,^50^,^51^ is the core element underlying envy during social comparison. Therefore, envy is likely to result from a circumstance in which a person has some trouble understanding an individual’s intention or the general situation, or perceives a lack of control or injustice. Thus, we conclude that the association between increased dispositional envy and higher GMV in the STG may indicate a decrease in social adaptation ability with regard to interpersonal communication.

In conclusion, the present study successfully identified potential neural correlates of dispositional envy using a VBM approach. Our results demonstrate that rGMV in the DLPFC and STG can be used to predict individual differences in dispositional envy: The former is implicated in emotional regulation, while the latter is involved in social reasoning and social perception, both of which may significantly influence the experience of envy. Finally, we revealed that emotional intelligence is an important mediator of the relationship between GMV in the DLPFC and dispositional envy, improving the current understanding of the complex relationship among emotional intelligence, dispositional envy, and brain activity. In a word, the present study is conductive to further understand the cognitive processing of envy, especially dispositional envy.

## Methods

### Participants and procedures

In the present study, we utilized two samples of data from a single group of healthy volunteers. Sample 1 included data from 73 participants (32 men, 41 women; mean age = 21.47 ± 2.08 years; age range: 18–26 years), while Sample 2 consisted 27 participants from the same population (11 men, 16 women; mean age = 20.63 ± 2.15; age range: 18–26 years). All participants were recruited from South China Normal University in Guangzhou, China. All participants were Chinese and right-handed with normal or corrected-to-normal vision. None of the participants had a history of mental or neurological illness. Written informed consent was obtained from all participants prior to the initiation of the research. This study was approved by and conducted in accordance with the Imaging Center Institutional Review Board of South China Normal University.

All participants first underwent an MRI scan during which they were instructed to refrain from head movement and remain awake. The scan was comprised of anatomical imaging (5 min) and resting state imaging (8 min). In this study, only anatomical imaging data was used. Participants were then asked to complete paper-and-pencil questionnaires including the Emotional Intelligence Scale and Dispositional Envy Scale, among others. Each participant took approximately 40 minutes to complete the study.

### Measures

#### Dispositional Envy Scale

The Dispositional Envy Scale (DES) is an eight-item self-report questionnaire designed to assess individual differences in envious tendencies^7^. Example statements include the following: *I feel envy every day* and *feelings of envy constantly torment me*. Each item is scored on a 5-point Likert-type scale, in which 1 = *strongly disagree*, 2 = *moderately disagree*, 3 = *neither agree nor disagree*, 4 = *moderately agree*, and 5 = *strongly agree*. Previous studies have demonstrated that this scale, which has been widely utilized to assess envy in a variety of populations, has high reliability^52^,^53^, with a Cronbach’s α of 0.88 in the present study.

#### Wong and Law Emotional Intelligence Scale (WLEIS)

The Wong and Law Emotional Intelligence Scale (WLEIS)^54^ is designed to assess emotional intelligence across the following four domains: Regulation of Emotion (ROE), Use of Emotion (UOE), Self Emotion Appraisals (SEA), and Others’ Emotion Appraisals (OEA). Participants were instructed to indicate the extent to which they agreed or disagreed with each statement using a 7-point Likert scale. Higher scores are indicator of greater emotional intelligence. This scale has been widely used in Chinese populations and has been reported to be a reliable and valid measure of emotional intelligence^55^,^56^,^57^,^58^. Total scores are used as an index of emotional intelligence. The Cronbach’s α was 0.90 in the present study.

#### MRI data acquisition

A 3.0 T Siemens Trio MRI scanner with a 12–channel head coil was used to acquire MR images. A three-dimensional magnetization-prepared rapid gradient-echo (3D MP-RAGE) sequence was used to obtain high-resolution T1-weighted anatomical images (repetition time (TR)/echo time (TE) = 1900 ms/2.52 ms, flip angle = 9°, resolution matrix = 256 × 256, FOV = 230 × 230 mm^2^, Slice thickness = 1.0 mm, Voxel size = 1 × 1 × 1 mm^3^).

#### Voxel-based morphometry

MR images were processed using SPM8 (Statistical Parametric Mapping, Wellcome Department of Imaging Neuroscience, London, UK) implemented in Matlab 10.0 (MathWorks Inc., Natick, MA, USA). Each image was first displayed in SPM8 to screen for artifacts or gross anatomical abnormalities. In the process of registration, the manual method was used to reorient the images to the anterior commissure. T1-weighted anatomical images were segmented into gray matter, white matter, and cerebrospinal fluid. We then performed diffeomorphic anatomical registration through exponentiated Lie algebra (DARTEL) in SPM8 for registration, normalization, and modulation of the data^59^. Gray matter images were rigidly aligned and resampled to 1.5 × 1.5 × 1.5 mm^3^ and normalized to a template in MN1152 space. Subsequently, an 8-mm full width at half-maximum Gaussian kernel was used to smooth the registered images. Finally, using absolute masking with a threshold of 0.2, the modulated images were masked to exclude noisy voxels.

#### Statistical analysis

Statistical analyses of gray matter volume (GMV) were performed using SPM8. In order to identify brain regions in which regional GMV was associated with individual differences in envy scores, a linear regression analysis was performed using DES score as the variable of interest. To control potentially confounding variables, we used age, sex, and total GMV as covariates in the regression model. In addition, The AlphaSim program was used to correct for multiple comparisons in AFNI (10,000 iterations) using REST software. The smoothing kernel was caluculated with 3dFWHMx. The new reestimated size of spatial smoothness was larger than orginal (original: 8, 8, 8 mm; new: 13.46, 13.79, and 13.09 mm). Using the new smooth size for multiple comparison correction, the voxel-wise intensity threshold was set at p < 0.001, and a cluster threshold of P < 0.05 (Cluster size ≥329) was set. AlphaSim has been widely used in previous VBM studies^55^,^60^,^61^,^62^.

After identifying potential neural bases of dispositional envy, we went on to investigate how emotional intelligence may affect the relationship between envy and correlated brain structures. To confirm whether emotional intelligence mediated the relationship between dispositional envy and these brain regions, a PROCESS macro program was utilized^63^,^64^.

#### Test-validation procedure

To ensure the validity of the test results, an additional 27 samples were collected from the same population according to the following process. We first defined the ROIs based upon the results obtained from the original 73 participants and extracted rGMV values for these ROIs for the 27 participants. We then performed multiple linear regression analysis in order to confirm whether rGMV of ROIs from the independent sample also predicted dispositional envy.

## Additional Information

**How to cite this article:** Xiang, Y. *et al*. Examining brain structures associated with dispositional envy and the mediation role of emotional intelligence. *Sci. Rep.* **7**, 39947; doi: 10.1038/srep39947 (2017).

**Publisher's note:** Springer Nature remains neutral with regard to jurisdictional claims in published maps and institutional affiliations.

## Figures and Tables

**Table 1:** Descriptive statistics for DES and WLEIS scores (n = 73).

	Means (SD)	Range	Skewness	Kurtosis	Correlation with Envy
DES	17.96 ± 8.35	8–39	0.52	−0.03	/
WLEIS	81.82 ± 12.64	49–103	−0.57	−0.58	−0.58***

Note: ***p < 0.001. DES: Dispositional Envy Scale; WLEIS: Wong and Law Emotional Intelligence Scale.

**Table 2:** Brain regions exhibiting significant correlation between GMV and DES scores.

Brain regions	Hemisphere	MNI coordinates			Number of voxels	Peak T-Value
		x	y	z		
STG	L	−51	4	3	508	4.123*
DLPFC	L	−19	49	24	396	4.400*

Note: GMV: gray matter volume; STG: superior temporal gyrus; DLPFC: dorsolateral prefrontal cortex; DES: Dispositional Envy Scale; MNI: Montreal Neurological Institute. *p < 0.05.
